# Silk/Polyamidoamine Membranes for Removing Chromium VI from Water

**DOI:** 10.3390/polym15081871

**Published:** 2023-04-13

**Authors:** Paolo Ferruti, Jenny Alongi, Emanuele Barabani, Amedea Manfredi, Elisabetta Ranucci

**Affiliations:** Dipartimento di Chimica, Università degli Studi di Milano, Via C. Golgi 19, 20133 Milano, Italy

**Keywords:** silk, polyamidoamine, composite hydrogels, chromium (VI), water purification

## Abstract

Polyamidoamine hydrogels prepared by the radical post-polymerization of α,ω-bisacrylamide-terminated M-AGM oligomers, in turn obtained by the polyaddition of 4-aminobutylguanidine with *N,N’*-methylenebisacrylamide, were reinforced with raw silk fibers, which can establish covalent bonds with the polyamidoamine matrix via reaction of the amine groups in the lysine residues with the acrylamide terminals of the M-AGM oligomer. Silk/M-AGM membranes were prepared by impregnating silk mats with M-AGM aqueous solutions and subsequent crosslinking by UV irradiation. The guanidine pendants of the M-AGM units imparted the ability to form strong but reversible interactions with oxyanions, including the highly toxic chromate ions. The potential of the silk/M-AGM membranes to purify Cr(VI)-contaminated water down to the drinkability level, that is, below 50 ppb, was tested by performing sorption experiments both in static (Cr(VI) concentration 20–2.5 ppm) and flow conditions (Cr(VI) concentration 10–1 ppm). After static sorption experiments, the Cr(VI)-loaded silk/M-AGM membranes could easily be regenerated via treatment with a 1 M sodium hydroxide solution. Dynamic tests performed using two stacked membranes and a 1 ppm Cr(VI) aqueous solution reduced Cr(VI) concentration down to 4 ppb. Remarkably, the use of renewable sources, the environmentally friendly preparation process, and the goal achieved meet eco-design requirements.

## 1. Introduction

Chromium ions are found in nature in two stable oxidation states: Cr(III) and Cr(VI). In aquifers, Cr(VI) is thermodynamically favored under oxidizing conditions and is highly soluble in water. In the absence of ligands other than OH- and H_2_O, HCrO_4_^−^ and CrO_4_^2−^ oxyanions are the most stable Cr(VI) species in groundwaters [1]. The occurrence of Cr(VI) in the environment is largely due to industrial activities for multiple purposes, such as manufacturing of anti-corrosion coatings, steel, and pigments, leather tanning, and wood preservation [2]. Regardless of anthropogenic activities, the release of Cr(VI) into waters can be caused by the erosion of rocks belonging to the spinel group that are present in ophiolitic sequences and related soils [3].

While Cr(III) is beneficial for the development of living organisms at trace amounts [4,5], Cr(VI) is carcinogenic to humans and is classified as a class A pollutant. According to the U.S. Department of Health and Human Service, the cancer potency of Cr(VI) is 0.5 mg^−1^ kg^−1^ day^−1^ [4]. Therefore, Cr(VI) contamination of groundwaters and soils represents a severe hazard for both the biota and human health. In Europe, the drinking water limit for total chromium concentration is 50 μg L^−1^, as stated in European Council Directive 98/83/EC (CoE, 1998), whereas the World Health Organization suggests 50 μg L^−1^ for Cr(VI) [6]. Moreover, regarding Italian regulations, the ministerial decree of 14 November 2016 introduces the limit of 10 μg L^−1^ of Cr(VI) in drinking water [7].

Currently, the most common remediation strategies for Cr(VI)-contaminated waters include activated carbon [8,9,10], reactive barriers [11], ion exchange [12,13,14,15,16], electrochemical precipitation [17], reverse osmosis [18], chemical precipitation [19], adsorption, and combined reduction–coagulation–filtration [20]. In the last decade, polymeric materials [21,22,23], composites [24,25,26], and nanocomposites [27,28] have also been proposed for this purpose. In addition, several side product deriving from agriculture proved to be efficient low-cost adsorbents for the removal of Cr(VI) from water, among which lignocellulosic residues were highly considered [29,30,31]. However, most of them show poor adsorption efficiency or require complex and expensive preparation processes. Therefore, the preparation of an adsorbent material with high performance towards Cr(VI) ions is desirable. From the material design viewpoint, adsorption capacity strongly depends on the density of the adsorption sites. In this regard, amine groups have been extensively studied for their strong interactions with Cr(VI) ions [32,33,34]. Several amine-functionalized adsorbents have been developed, among them composite adsorbents combining PAMAM dendrimers with organic [35,36,37] or inorganic solids [38,39,40]. However, all these treatments are not always sufficiently efficient to satisfy the severe requirements for drinking water. Therefore, it would be of paramount importance to develop materials for the removal of Cr(VI) down to the threshold values required by regulations [7].

The guanidinium ion has wide potential in the recognition of anions [41]. Furthermore, the guanidine moiety of arginine is responsible for the strong arginine–phosphate interactions of proteins [42]. Thanks to its peculiar molecular geometry, charge distribution, and hydrogen bonding ability, the guanidinium ion can indeed strongly bind bidentate oxyanions, including, inter alia, carboxylate, phosphate, sulfate, nitrate, and selenate [43,44,45,46,47,48,49]. Unsurprisingly, it has been reported that polyhexamethylene–guanidine modified natural zeolitic materials [50], and guanidinium-based ionic organic covalent nanosheets [51,52] uptake considerable amounts of Cr(VI) oxyanions from aqueous solutions.

Polyamidoamines (PAAs) are multifunctional polymers synthesized by the aza-Michael polyaddition of prim-monoamines or sec-diamines with bisacrylamides [53,54]. The reaction meets many of the requirements of green chemistry as it takes place in water at room temperature in the absence of organic solvents or added catalysts. PAA synthesis can involve many conceivable amines, including the amine groups of natural amino acids and bisacrylamides. Even the residual amine groups of proteins can participate in the reaction, leading to hybrid PAA–protein graft copolymers [55]. The guanidine group, well known for providing stable interactions with oxyanions, can be inserted as pendants in PAAs by using 4-aminobutyl guanidine (agmatine) as a co-monomer [56] since agmatine can be made to react with bisacrylamides only through the primary amine groups and proper adjustment of the pH of the reaction mixture [57]. Many PAAs have proven capable of providing stable complexes with heavy metal ions such as Cu(II), Ni(II), Co(II), and Mn(II), both as linear soluble polymers [58] and crosslinked resins [59,60]. The PAAs carrying guanidine groups can be expected to establish strong interactions with oxyanions, including chromate groups.

Different approaches can be adopted to obtain crosslinked PAAs. A direct one-pot procedure could include employing multifunctional amines as crosslinking agents. A second equally useful approach is first synthesizing an acrylamide-terminated PAA precursor and then photopolymerizing it via UV irradiation, a commonly used procedure thanks to its wide industrial applicability and great potential in the production of added-value materials, including lithography, adhesives, coatings, and 3D printing, just to mention a few [61]. It may be observed that, in this process, the acrylamide terminals of the oligomeric PAA precursors are still susceptible to undergoing aza-Michael addition reaction with amines. In a previous work, silk mats obtained from degummed raw silk fibers proved capable of reinforcing agmatine-derived PAA hydrogels by giving rise to covalent bonds with the polymer matrix, likely thanks to reaction of the amine groups derived from the lysine residues with activated α,ω-acrylamide terminals of the oligomeric precursors in the final crosslinking step [62].

According to this premise, silk-reinforced PAA hydrogel membranes bearing guanidine pendants in the repeat units were studied in this work for their potential to remove Cr(VI) from contaminated waters due to their ability to provide strong but reversible interactions with chromate ions.

## 2. Materials and Methods

### 2.1. Materials

Lithium hydroxide monohydrate (≥98%) and sodium bicarbonate (≥99.0%) were supplied by Fluka (Milano, Italy). 4-Aminobutylguanidine sulphate (agmatine, AGM, 95%) was supplied by Enamine (Riga, Latvia). Fuming hydrochloric acid (≥37% in water), 1 M sulfuric acid, acetone (99.5%), *N,N’*-methylenebisacrylamide (MBA, 99%), potassium chromate (≥99.0%), glacial acetic acid (≥99%), and nitric acid (65-67%) were supplied by Sigma Aldrich (Milano, Italy) and used as received. Raw Silk Hankies obtained from a degummed cocoon were supplied by Beesybee Fibers (Bolinas, CA, USA). A stainless-steel wire mesh sieve (mesh 60, 250 μm) was purchased from Giuliani Tecnologie s.r.l. (Torino, Italy).

### 2.2. Methods

The chemical structure of the oligomeric M-AGM precursor was confirmed by ^1^H Nuclear Magnetic Resonance (^1^H-NMR), collecting spectra in D_2_O at 25 °C using a Bruker Avance DPX-400 NMR spectrometer (Milano, Italy) operating at 400.13 MHz.

The photo-polymerization reaction was performed using a UV-A (315–400 nm) lamp HG 200 ultra with 250 W power supplied by Jelosil s.r.l. (Vimodrone, Milano, Italy).

The surface morphology of the silk mats and silk/M-AGM membranes was analyzed using a Hitachi TM-1000 scanning electron microscope (SEM) (Oyama, Japan) operating under 15 kV beam voltage. Membrane samples (5 mm × 5 mm) were fixed to the sample holder through a conductive carbon adhesive tape.

The dimensions of the processed silk fibers, as well as the thickness of both the silk mats and the silk/M-AGM membranes, were measured with a Dino-Lite Edge digital microscope (AM7115MZT model) with 5-megapixel resolution (VWR International s.r.l., Milano, Italy).

### 2.3. Silk Mat Preparation

Silk chops 10 mm × 5 mm in size were obtained by cutting degummed raw silk (1.0 g). They were then suspended in ultrapure water (400 mL) and ground for 5 min at 25 °C with an Ultra-Turrax^®^ IKA T25 disperser (IKA, Staufen, Germany) with a 22–23 × 10^3^ rpm spinning rate, obtaining a slurry of silk fibers 0.3–2.0 mm in length and 5–15 µm in diameter. The dimensions of the silk fibers were determined by placing 5 drops of the processed silk mash on a glass petri dish, and water was removed via heating with a stream of hot air. Individual silk fibers laying on the glass surface were then analyzed by digital microscopy. The silk slurry was then vacuum-filtered through a stainless-steel wire mesh sieve with 250 μm pore size. A circular silk mat was obtained (10 cm in diameter, 236 ± 40 μm in thickness) that was dried in a hot air stream (100 °C × 5 min per side). Yield: 0.6 g (60% yield). Discs of 2.5 and 7.5 cm in diameter were cut and weighed (33.1 ± 6.9 mg and 367.2 ± 32.3 mg, respectively).

A second series of silk mat samples was prepared by following the same procedure; however, before cutting, the 10 cm discs were compressed through two counter-rotating rollers, giving slightly larger discs with an average thickness 139 ± 10 μm. Discs of 2.5 cm in diameter were cut and weighed (45.8 ± 3.6 mg).

### 2.4. Synthesis of the α,ω-Bisacrylamide-Terminated M-AGM Oligomer

M-AGM was synthesized as already reported [56]. Briefly, the reactive solution was obtained by mixing *N,N’*-methylenebisacrylamide (7.00 g; 0.045 mol), agmatine sulphate (8.64 g; 0.038 mol), and lithium hydroxide monohydrate (1.56 g; 0.038 mol) in water (15 mL). This solution was heated to 40–45 °C until completely dissolved and then left for 5 days at room temperature in the dark. A 1 mL portion of the solution was acidified to pH 4.5 using 6 M HCl and freeze-dried, and the resulting product was analyzed by ^1^H-NMR. The remaining solution was used in the preparation of the M-AGM hydrogel.

### 2.5. Synthesis of the Silk/M-AGM Membranes

Different sets of 130 μm thick silk/M-AGM membranes were prepared by adopting three slightly different preparation procedures (A, B, and C). These procedures will be reported separately.

Procedure A: Silk/M-AGM membranes in the form of 130 μm thick discs with a diameter of 2.5 cm were obtained in a glass mold made by overlapping two silanized 2 mm thick glass sheets intercalated by a 130 μm thick Parafilm^®^ frame with a circular gap 2.5 cm in diameter. The glass plates had been previously washed by soaking them in aqua regia for 5 h, were then rinsed with water, dried, and finally silanized with trichloromethylsilane vapors in a closed chamber for 3 days. After silanization, the plates were washed with toluene (20 mL), ethanol (2 × 20 mL), and water (3 × 20 mL) and were then finally gently wiped.

The silk/M-AGM membranes were synthesized by first placing a portion of the α,ω-bisacrylamide-terminated M-AGM oligomer solution (see Section 2.4) (0.15 mL) in the open mold inside the frame. A 2.5 cm diameter disc of compressed silk mat (approximately 30 mg and 139 μm thickness in dry form) was then inserted in the mold. An additional portion of the M-AGM solution (0.15 mL) was subsequently added and the mold was closed with a second glass plate and blocked with metal clips, taking care to let out the excess M-AGM solution to ensure complete silk impregnation. The crosslinking reaction was subsequently triggered via UV irradiation of both mold sides with a UV-VIS lamp (315–400 nm emission range, 15 cm distance, and 30 min per side). The silk/M-AGM membrane was finally retrieved from the mold and dried overnight. Samples obtained by this procedure are coded as silk/M-AGMA2.5, where A stands for the adopted procedure and 2.5 for the diameter. Their characteristics are shown in Table 1.

Procedure B: Silk/M-AGM membranes in the form of 130 μm thick discs with a diameter of 2.5 or 7.5 cm were obtained as described in procedure A except with the use of uncompressed silk mats of suitable size (30 or 330 mg, respectively, and 236 ± 40 μm thickness). After the crosslinking step, the samples were retrieved from the mold and dried overnight. Samples obtained by this procedure are coded as silk/M-AGMB2.5 or silk/M-AGMB7.5, where B stands for the adopted procedure and 2.5 and 7.5 stand for the diameter. Their characteristics are shown in Table 1.

Procedure C: Silk/M-AGM membranes in the form of 130 μm thick discs with a diameter of 2.5 or 7.5 cm were obtained as described in procedure B up to the crosslinking step. Afterwards, the samples were retrieved and soaked in a 20 wt.% acetic acid aqueous solution (50 or 200 mL for 2.5 or 7.5 cm membranes, respectively) for 4 h, dried over calcium chloride, soaked in a 3 wt.% sodium bicarbonate aqueous solution (50 or 200 mL for 2.5 or 7.5 cm membranes, respectively) for 6 h, and dried again overnight over sodium hydroxide. Finally, the membranes were washed with distilled water to neutral pH and dried to constant weight. Samples obtained by this procedure are coded as silk/M-AGMC2.5 or silk/M-AGMC7.5, where C stands for the adopted procedure and 2.5 and 7.5 stand for the diameter. Their characteristics are shown in Table 1.

### 2.6. Water Uptake Tests

Water uptake tests were performed on silk/M-AGM membranes with dimensions of 25 mm × 10 mm and 0.15 mm (40 mg). They consisted of water absorption/drying cycles (each carried out by first soaking the membranes in ultrapure water for 8 h), gentle blotting with paper towels, and then weighing. The swollen samples were then dried for 48 h at 25 °C in a drying chamber containing solid calcium chloride as desiccant. The water uptake of the membranes (WU%) was calculated by Equation (1):

(1) $$WU\%=\frac{W_{t}-W_{o}}{W_{o}}\times 100$$

where W_o_ = weight of the dry hydrogel and W_t_ = weight of the swollen sample.

### 2.7. Determination of Cr(VI) Concentrations

The Cr(VI) concentrations of aqueous solutions from 10 to 1 ppm were determined by UV-VIS spectroscopy using a Jasco V-730 spectrophotometer (Milano, Italy) operating at 349 nm and pH 3.0, while those below 1 ppm were determined by atomic absorption spectroscopy in 2% *v/v* nitric acid solution using a Perkin-Elmer PinAAcle 900T Spectrometer (Milano, Italy).

The UV-VIS spectrophotometer was calibrated according to a standard procedure [63] using Cr(VI) aqueous standard solutions with concentrations of 1.0, 5.0, 10.0, 15.0, 20.0, and 30.0 ppm at pH 3.0. In detail, a 100 ppm Cr(VI) stock solution was first prepared by dissolving 93.3 mg K_2_CrO_4_ in 250 mL of water in a volumetric flask. The aqueous standard solutions were then obtained by adding appropriate amounts of the stock solution (1, 5, 10, 15, 20, and 30 mL, respectively) to a 100 mL volumetric flask, with 1 mL of 1 M sulfuric acid also added before water was finally used to fill to the mark. All measurements were performed at 349 nm, corresponding to the maximum absorbance of HCrO_4_^−^, which is the prevailing species in the pH range 2–4 [64]. The calibration equation obtained was as follows:

(2) A=0.0299×C

where *A* is the absorbance of the calibrant solution and *C* is the Cr(VI) concentration of the calibrant solution expressed in ppm. The value of the proportionality constant of Equation (2), determined as the slope of the straight line passing through the origin of the Cartesian axes and obtained by plotting the set of experimental data *A* vs. *C*, corresponded to a linear regression coefficient (R^2^) of 0.9999.

The atomic absorption spectrophotometer was calibrated using Cr(VI) solutions with concentrations of 0.05, 0.1, 0.25, 0.5, and 1.0 ppm. In detail, an initial 1000 ppm Cr(VI) stock solution was prepared by dissolving 934.0 mg K_2_CrO_4_ in 2% *v/v* nitric acid water solution in a 250 mL volumetric flask. A second 10 ppm Cr(VI) stock solution was obtained by diluting 1 mL of the first stock solution with 2% *v/v* nitric acid in a in 100 mL volumetric flask.

The final Cr(VI) water solutions were prepared in a 100 mL volumetric flask by adding appropriate amounts of the 10 ppm stock solution (0.5, 1, 2.5, 5.0, and 10.0 mL, respectively) and filling to the mark with a 2% *v/v* nitric acid solution.

Two valid calibration curves were obtained in the 0.05–0.25 ppm and 0.25–1.0 ppm ranges, respectively.

The calibration equations obtained were as follows:

(3) A=0.0414×C

(valid in the 0.05–0.25 ppm range)

(4) A=0.0352×C

(valid in the 0.25–1.0 ppm range)
where *A* is the absorbance of the calibrant solution and *C* is the Cr(VI) concentration of the calibrant solution expressed in ppm. The values of the proportionality constant of Equations (3) and (4), determined as the slopes of the straight line passing through the origin of the Cartesian axes and obtained by plotting the set of experimental data *A* vs. *C*, corresponded to linear regression coefficients (R^2^) of 0.9998 and 0.9954, respectively.

### 2.8. Cr(VI) Sorption Tests

Sorption tests were performed under both static and dynamic conditions. Before sorption tests, dry silk/M-AGM membranes were conditioned in distilled water for 10 min and then gently wiped with filter paper.

#### 2.8.1. Cr(VI) Sorption in Static Conditions

Static Cr(VI) sorption tests were carried out on silk/M-AGMA2.5 samples (Table 1). In these experiments, a 2.5 cm diameter membrane was incubated in 5 mL of a Cr(VI) aqueous solution (2.5, 5, 10, and 20 ppm) at pH 7.0 for 24 h. After this time, the membrane was retrieved, the solution acidified to pH 3.0 with 1 M sulfuric acid, and the residual Cr(VI) concentration determined by UV-VIS spectroscopy at 349 nm. All tests were performed at least in triplicate.

#### 2.8.2. Cr(VI) Sorption under Flow Conditions

Dynamic Cr(VI) sorption tests were carried out on silk/M-AGMB2.5, silk/M-AGMB7.5, silk/M-AGMC2.5, and silk/M-AGMC7.5 samples (Table 1) by running ultrafiltration experiments in dead-end mode under controlled transmembrane pressure conditions (2.5 atm). Silk/M-AGM membranes were placed in an AMICON^®^ (Millipore, Milano, Italy) ultrafiltration apparatus of suitable size, i.e., 50 mL for 2.5 cm diameter membranes and 600 mL for 7.5 cm diameter membranes. The feed reservoir was then filled with Cr(VI) aqueous solutions with a known concentration, that is, 30 mL of a 10 ppm Cr(VI) solution for 2.5 cm diameter membranes and 300 mL of a 1 ppm Cr(VI) solution for 7.5 cm diameter membranes.

In the initial set of experiments, regardless of the starting Cr(VI) concentration and membrane diameter, the starting solution was ultrafiltered four times using the same membrane. After each filtration step, an aliquot of the ultrafiltered solution was acidified to pH 3.0 and the residual Cr(VI) concentration measured (see Section 2.7).

A second set of experiments was performed by ultrafiltering 10 ppm Cr(VI) solutions four times through four stacked membranes 2.5 cm in diameter separated by plastic mesh discs. A third set of experiments was performed by ultrafiltering 1 ppm Cr(VI) solutions four times through two stacked membranes 7.5 cm in diameter separated by a plastic mesh disc. After each filtration step, an aliquot of the ultrafiltered solution was acidified to pH 3.0 and the residual Cr(VI) concentration measured (see Section 2.6). All tests were performed at least in triplicate.

### 2.9. Regeneration Tests

Regeneration tests were carried out on Cr(VI)-loaded silk/M-AGMA2.5 membranes following sorption experiments performed by incubating these membranes in 5 mL of either a 20 ppm or a 10 ppm Cr(VI) solution for 24 h under static conditions. The same sorption tests were repeated on Cr(VI)-loaded silk/M-AGMB7.5 membranes using 10 ppm Cr(VI) solutions under the same conditions.

Regeneration tests were performed by soaking wet Cr(VI)-loaded 2.5 cm diameter membranes in 5 mL of a 1 M sodium hydroxide solution under static conditions for 24 h. Alternatively, 7.5 cm membranes were soaked in 20 mL of 1 M sodium hydroxide under the same conditions. After this time, the membranes were removed, the solution was acidified to pH 3.0 with 1 M sulfuric acid, and the amount of released Cr(VI) was determined by UV-VIS spectroscopy at 349 nm.

## 3. Results and Discussion

### 3.1. Rationale

Cr(VI) anions are known to strongly interact with guanidinium cations through a combination of ionic and hydrogen bonding [41,42,43,44,45,46,47,48,49,50,51,52]. Theoretical studies have indeed demonstrated that the hydrogen bond interactions between the bidentate guanidinium and oxyanion-charged species produce very strongly bound complexes [48]. In a recent paper [62], it was demonstrated that polyamidoamine (PAA) hydrogels can be remarkably toughened by embedding a silk mat prepared from short silk fibers (0.3–2.0 mm) that is capable of establishing covalent bonds with the PAA matrix without any specific chemical treatment. This suggested that by reinforcing a purposely designed guanidinium-containing PAA hydrogel with silk, the resultant composite combined reasonable mechanical strength with the ability to act as effective Cr(VI) sorbent for the purification of Cr(VI)-contaminated water.

**Figure 1:**
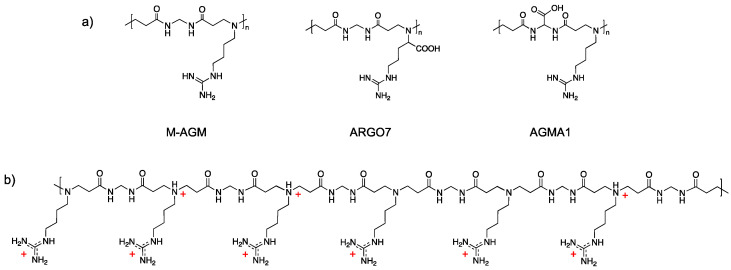
(**a**) M-AGM, ARGO7, and AGMA1 repeat units; (**b**) M-AGM ionic species distribution.

The PAA coded M-AGM (Figure 1a), bearing in its repeating unit a tert-amine group and a guanidine pendant, has optimal structural requirements to efficiently interact with chromate anions. In fact, its ionic charge distribution at pH 7 (Figure 1b) is characterized by 50% protonated tert-amine groups in the backbone (tert-amine *pKa* = 7.1) and 100% protonated guanidine groups (guanidine *pKa* > 10) [56]. Again, at pH 7, at which Cr(VI) sorption tests have been performed, both the HCrO_4_^−^ and CrO_4_^2−^ species are present, the HCrO_4_^−^/CrO_4_^2−^ equilibrium being 80% shifted towards the HCrO_4_^−^ form [64]. The Cr_2_O_7_^2−^ species achieves its maximum concentration (10%) in the 2–4 pH range and is virtually absent at pH 7. Both HCrO_4_^−^ and CrO_4_^2−^ can, in principle, ionically interact with both the ammonium ions in the backbone and the guanidinium pendants of M-AGM. However, as stated above, it is generally recognized that the bidentate interactions of guanidinium ions with oxyanions are very strong and prevail over interactions with the simple ammonium ions.

It should be observed that the M-AGM structure guarantees optimal Cr(VI) sorption efficacy, since PAA hydrogels of related structures bearing a guanidine pendant for repeat unit, but containing also a repulsive carboxylate anion, are not equally efficient in ionically interacting with Cr(VI) anions. Notably, both the PAAs coded ARGO7 and AGMA1 (Figure 1a) bearing a carboxylate group in the amine moiety and in the amide moiety, respectively, proved in preliminary studies to be poor Cr(VI) sorbents.

The synthetic procedure adopted for obtaining silk-reinforced M-AGM membranes consisted of first preparing a solution of an α,ω-acrylamide-terminated M-AGM oligomer, impregnating it with a silk mat, and finally inducing radical polymerization of the vinyl terminals through UV irradiation in a mold. Covalent bonds between silk and the hydrogel matrix were established via aza-Michael reaction of the amine residues present in the lysine units of silk with a portion of the terminal acrylamide groups of the M-AGM precursor.

### 3.2. Silk Mat Preparation

**Figure 2:**
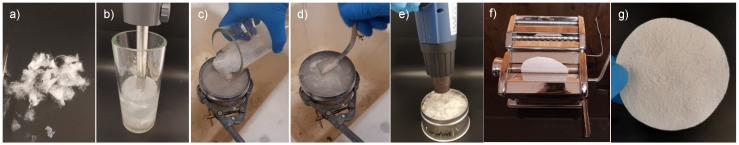
General scheme of the preparation of a short-fiber silk mat from cocoons: (**a**) chops of degummed silk; (**b**) grinding silk chops dispersed in water into short fibers (0.3–2 mm); (**c**) filtration of the ground silk dispersion through a 250 μm mesh sieve; (**d**) compression of the silk mash with a spatula; (**e**) drying of the silk mash under a hot air stream; (**f**) compression of the silk mat through counter-rotating rolls; (**g**) compressed mat.

The silk mat used as a PAA hydrogel reinforcer was obtained from degummed raw silk by first preparing a fine aqueous dispersion of short silk fibers with a homogenizer (0.3–2.0 mm in length and 5–15 µm in diameter, as assessed by optical microscopy analysis) and then filtering the resultant dispersion through a 250 μm stainless-steel mesh before finally drying the obtained solid cake under a hot air stream (Figure 2). Mats prepared by this procedure were used to synthesize silk/M-AGMB2.5, silk/M-AGMB7.5, silk/M-AGMC2.5, and silk/M-AGMC7.5 membranes (Table 1). In the synthesis of silk/M-AGMA2.5 samples, the dried mats were pressed through two counter-rotating rolls before impregnation with the reactive PAA oligomer (see Section 2.5). The silk mats resembled felt (Figure 2g).

**Figure 3:**
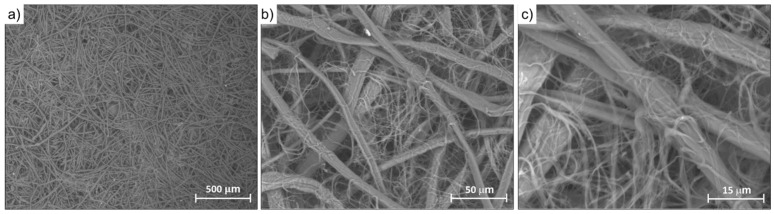
SEM micrographs of the silk mat at different magnifications ((**a**): 100×, (**b**): 1000×, (**c**): 2500×). The filaments with smaller diameter correspond to sericin residues, whereas the fibers with large diameter correspond to fibroin.

Scanning Electron Microscopy (SEM) analyses (Figure 3) showed a morphology consisting of interweaved fibroin fibers with some serine filaments. Noticeably, the silk mat was characterized by a porous structure that guaranteed integration with the M-AGM hydrogel.

### 3.3. Synthesis of Silk/M-AGM Membranes

**Figure 4:**
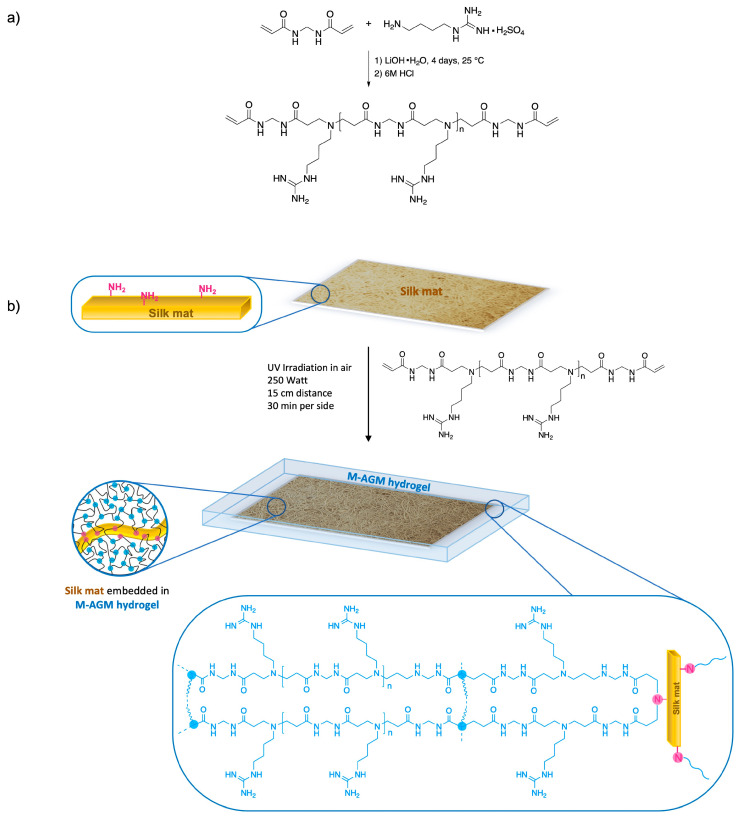
Synthesis of the silk/M-AGM hydrogel: (**a**) synthesis of M-AGM oligomer; (**b**) synthesis of silk/M-AGM hydrogel.

**Figure 5:**
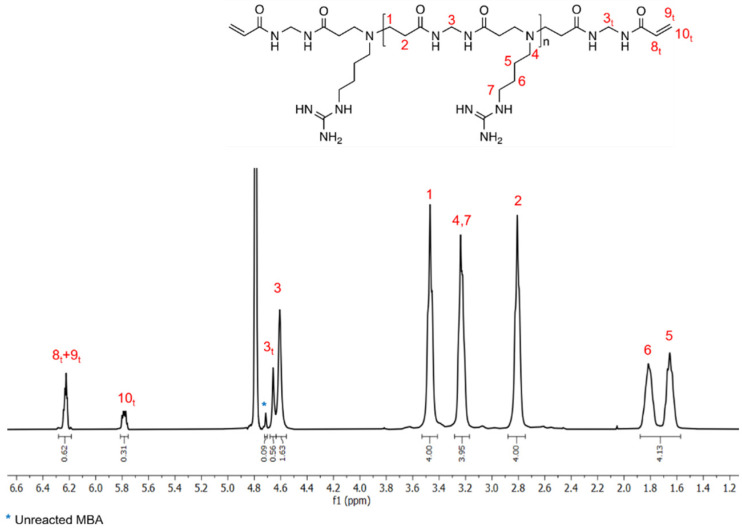
^1^H-NMR spectrum of the α,ω-bisacrylamide-terminated M-AGM oligomer run in D_2_O using a 400 MHz Brüker Avance spectrometer.

Synthesis of the silk/M-AGM membranes was carried out in three main steps. In particular, the first step consisted of the synthesis of an α,ω-acrylamide-terminated M-AGM oligomer susceptible to UV-induced radical crosslinking, which was obtained in water from a *N,N’*-methylenebisacrylamide/agmatine mixture at a molar ratio of 1.2/1.0 (Figure 4a). The structure of the M-AGM oligomer was determined by ^1^H-NMR spectroscopy (Figure 5).

The subsequent step consisted of impregnating the silk mat with an M-AGM oligomer solution and then triggering the radical polymerization of the terminal acrylamide groups (Figure 4b) inside a mold through UV irradiation. During this process, covalent bonds between the silk mat and hydrogel matrix were established via the aza-Michael reaction between the amino functions of the lysine residues and part of the terminal acrylamide groups of the α,ω-acrylamide-terminated M-AGM oligomers. The resultant silk/M-AGM membranes were recovered by gently detaching them from the mold and were subsequently extensively extracted with ultrapure water. They were flexible, pliable, and homogeneous and could be handled without being damaged. It should be observed here that plain M-AGM hydrogels of the same thickness as the silk/M-AGM membranes prepared by the described procedures (around 130 μm) were extremely brittle and could not withstand handling, whereas the silk-reinforced membranes could withstand prolonged compressive stresses during the ultrafiltration experiments (see Section 3.7).

The codes and compositions of all synthesized silk/M-AGM membranes are shown in Table 1. It may be observed that these membranes were obtained from silk mats prepared following two slightly different procedures (see Section 2.5) and were characterized by different average compositions and morphologies (see Section 3.4). In particular, the silk/M-AGM_A2.5_ samples were prepared from silk mats that, after filtration and drying, had been compressed through two counter-rotating rollers (Figure 2f). The remaining samples (silk/M-AGMB2.5, silk/M-AGMB7.5, silk/M-AGMC2.5, and silk/M-AGMC7.5) were prepared from silk mats that, after filtration and drying, had not been compressed. Among them, the silk/M-AGMB2.5 and silk/M-AGMB7.5 samples were not subjected to further chemical treatments after the crosslinking step, whereas the silk/M-AGMC2.5 and silk/M-AGMC7.5 samples were sequentially treated with an acetic acid and a sodium hydrogen carbonate solution. The average weight percentage of hydrogel in the silk/M-AGMA2.5 samples was lower than that of the silk/M-AGMB2.5 and silk/M-AGMB7.5 samples (Table 1) since, in the latter, prior to the crosslinking step, the dry uncompressed silk mats had been impregnated with larger amounts of the M-AGM oligomeric solution. It should be observed that the silk/M-AGMC2.5 and silk/M-AGMC7.5 samples had a hydrogel average weight percentage lower than that of silk/M-AGMB2.5 and silk/M-AGMB7.5 samples, notwithstanding they had been obtained from uncompressed silk mats; the percentages were comparable to those of the silk/M-AGMA2.5 samples. This was most probably a consequence of the acid/base treatment to which they had been subjected, causing carbon dioxide evolution with consequent mechanical stresses that probably resulted in partial material loss.

### 3.4. Morphological Characterization of Silk/M-AGM Membranes

**Figure 6:**
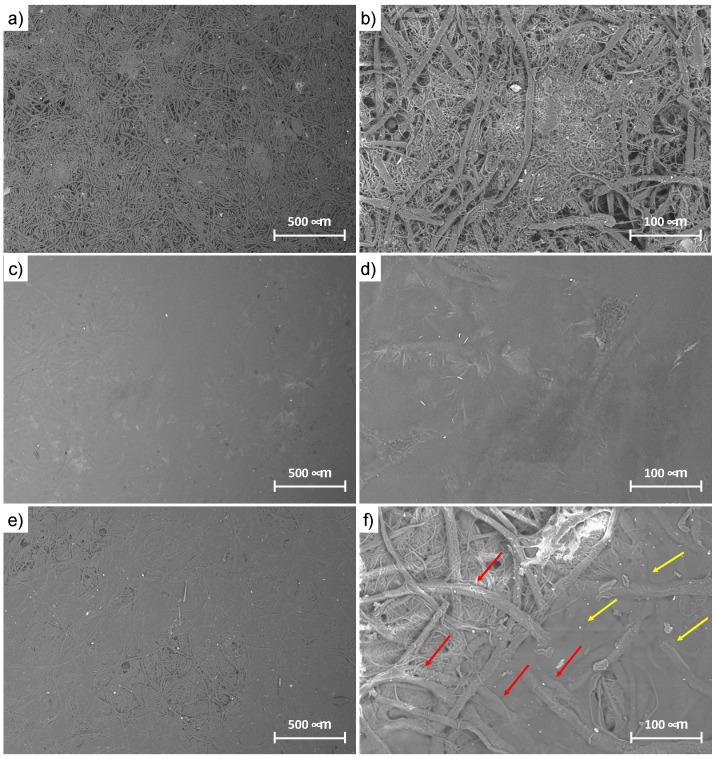
SEM micrographs of silk/M-AGM composite hydrogels at different magnifications: (**a**,**b**) silk/M-AGMA2.5; (**c**,**d**) silk/M-AGMB2.5; (**e**,**f**) silk/M-AGMC2.5. Yellow arrows: M-AGM hydrogel matrix; red arrows: silk fibers.

The morphology of the silk/M-AGM membranes was investigated by means of SEM (Figure 6). It is apparent that in the silk/M-AGMA2.5 samples, obtained from silk mats which had been subjected to compression treatment after filtration and drying, the silk fibers were homogeneously embedded in the M-AGM hydrogel matrix (Figure 6a). However, the silk fibers retained their individuality and had a somewhat porous structure that is even more evident in the magnified image (Figure 6b). Conversely, in the silk/M-AGMB2.5 samples obtained from silk mats that had not been subjected to compression treatment after filtration and drying and that had been impregnated with a larger amount of reactive oligomeric solution, the silk fibers were so integrated in the hydrogel (Figure 6c) that they formed a continuous structure in which no pores can even be identified in the magnified image (Figure 6d). However, in the silk/M-AGMC2.5 samples, which had been subjected to a post-synthesis chemical treatment with acetic acid and sodium hydrogen carbonate, a certain extent of porosity can be observed at higher magnification. As stated above, the M-AGM swollen matrix underwent internal carbon dioxide evolution during treatment with consequent mechanical stress and the creation of local gaps and holes (Figure 6e,f).

The porous structure of the silk/M-AGM membranes will obviously play a role in the uptake of Cr(VI). This turned out to be particularly evident in dynamic tests (see Section 3.7), in which membrane porosity influenced both the flow rate of the ultrafiltration process and the accessibility of the active sites in the sorbent.

### 3.5. Water Uptake of Silk/M-AGM Membranes

**Figure 7:**
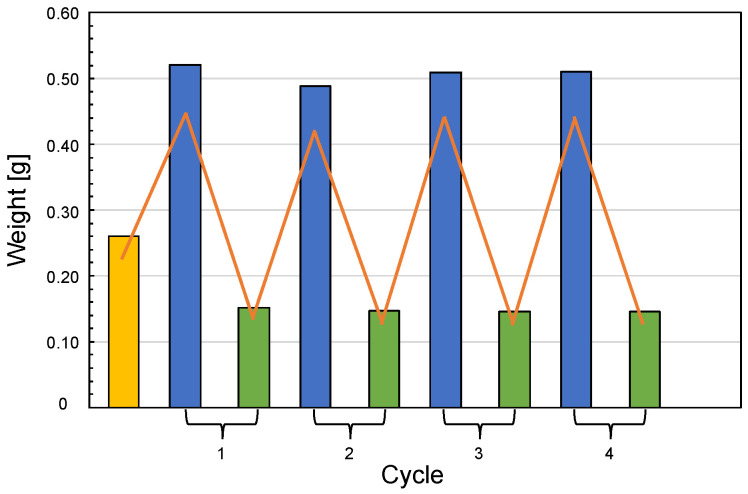
Water uptake of silk/M-AGM membranes. Yellow bar: sample just extracted from the mold; blue bars: samples swollen by incubation in distilled water for 8 h; green bars: residues left by the swollen samples after being dried and stored in a desiccator over solid calcium chloride for 24 h.

Figure 7 shows the results of the water uptake tests performed on silk/M-AGM samples. It is apparent that the swelling behavior was fully reversible after multiple swelling/deswelling cycles in water. Furthermore, all silk/M-AGM membranes could withstand several weeks immersed in water without any evidence of separation between the hydrogel and fibrous components. This indirectly confirms the presence of covalent bonds between them.

### 3.6. Cr(VI) Sorption Tests in Static Conditions

Cr(VI) sorption experiments were performed to study the ability of silk/M-AGM membranes to break down the amount of Cr(VI) in water to negligible quantities (i.e., <50 ppb) in both static and dynamic conditions, in particular determining their potential to make Cr(VI)-polluted water that already underwent purification treatment for industrial applications also drinkable or, in a broad sense, suitable for human use.

**Figure 8:**
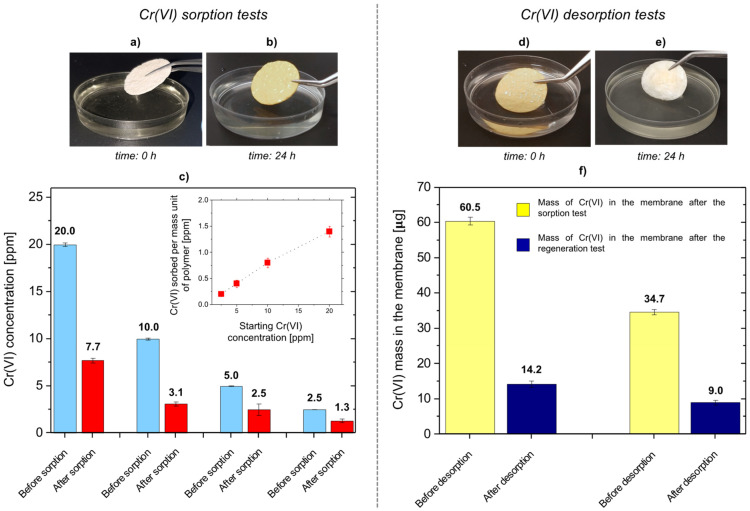
Cr(VI) sorption and desorption tests on silk/M-AGMA2.5 membranes in static conditions. **Left side**: Snapshots of a membrane at 0 and 24 h (panels **a** and **b**, respectively) of incubation in a 10 ppm Cr(VI) solution; results of the sorption tests carried out with Cr(VI) aqueous solutions at concentrations between 2.5 and 20 ppm (panel **c**). In panel (**c**), sky-blue bars represent the Cr(VI) concentration of the starting solution before sorption tests and red bars represent the Cr(VI) concentration of the solution after sorption tests. The insert represents the relationship between the amount of Cr(VI) sorbed per mass unit of M-AGM in the membrane and Cr(VI) concentration. **Right side**: Snapshots of a Cr(VI)-loaded silk/M-AGM membrane after incubation in a 10 ppm Cr(VI) solution (panel **d**), pictures of the same membrane after incubation in a 1 M NaOH solution (panel **e**), and results of the desorption tests on the silk/M-AGMA2.5 membranes previously subjected to static sorption tests in 20 ppm and 10 ppm Cr(VI) solutions after incubation in 1 M NaOH for 24 h (panel **f**). In panel (**f**), yellow bars represent Cr(VI) loading before desorption tests and blue bars represent Cr(VI) loading after regeneration.

Static sorption tests were carried out on silk/M-AGMA2.5 membranes that were incubated for 24 h in 5 mL Cr(VI) solutions with concentrations from 10 to 2.5 ppm, that is, well above the legal limits for potability [7]. The aim was to identify the dose-responsiveness of the silk/M-AGM membranes. The results of Cr(VI) sorption tests carried out in static conditions are shown in Figure 8 and Table 2. It should be noted that sorption tests performed on plain silk mats with 10 ppm Cr(VI) solutions have shown negligible sorption capacity.

It may be observed that the silk/M-AGM membranes showed significant Cr(VI) removal ability, ranging between 69% and 48% in the selected conditions (Table 2). Moreover, the amount of sorbed Cr(VI) per mass unit of the M-AGM hydrogel matrix was inversely proportional to the Cr(VI) concentration in the solution, clearly dependent on the law of mass action (Figure 8c). Percentagewise, slightly lower sorption was observed at 20 ppm compared to 10 ppm (Table 2), suggesting that the sorption phenomenon is gradually heading towards a plateau due to saturation of the sorbing medium.

The regenerability of the silk/M-AGM membranes was studied by incubating Cr(VI)-loaded membranes in 1 M NaOH for 24 h following static sorption tests carried out in 20 ppm and 10 ppm Cr(VI) solutions. Cr(VI) desorption was evident by discoloration of the Cr(VI)-loaded silk/M-AGM membranes (Figure 8d,e). The percentage of Cr(VI) released in both cases was comparable, being 72% and 74%, respectively (Figure 8f). These results, obtained through a single washing step carried out under static conditions, confirmed that the ionic interaction of the chromate anions with the pendant guanidinium groups of the M-AGM hydrogel is reversible. Therefore, it may be reasonably assumed that regeneration would proceed to a greater extent with repeated washing with alkaline solutions either in static or dynamic conditions.

### 3.7. Cr(VI) Sorption Tests under Flow Conditions

**Figure 9:**
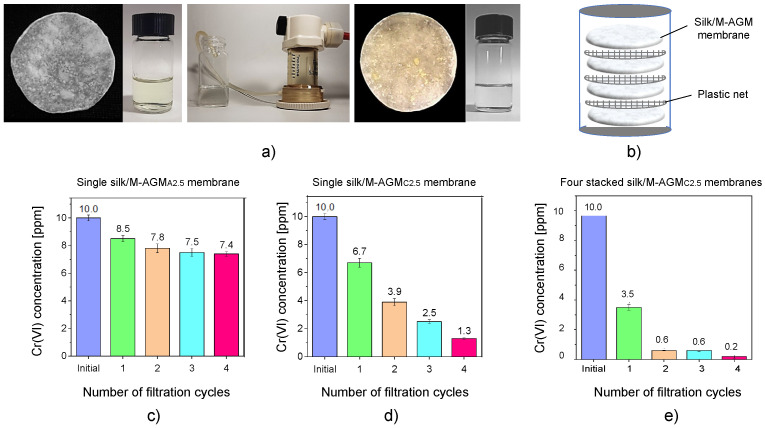
Dynamic sorption tests carried out with 10 ppm Cr(VI) solutions: pictures of (**a**) the starting Cr(VI) solution and silk/M-AGMA2.5 membrane before ultrafiltration, the ultrafiltration apparatus, and the final Cr(VI) solution and silk/M-AGMA2.5 membrane after ultrafiltration, and (**b**) the scheme of the ultrafiltration apparatus with four stacked membranes separated by plastic nets. Results of the sorption tests with (**c**) a single silk/M-AGM A2.5 membrane, (**d**) a single silk/M-AGMC2.5 membrane, and (**e**) four stacked silk/M-AGMC2.5 membranes.

**Figure 10:**
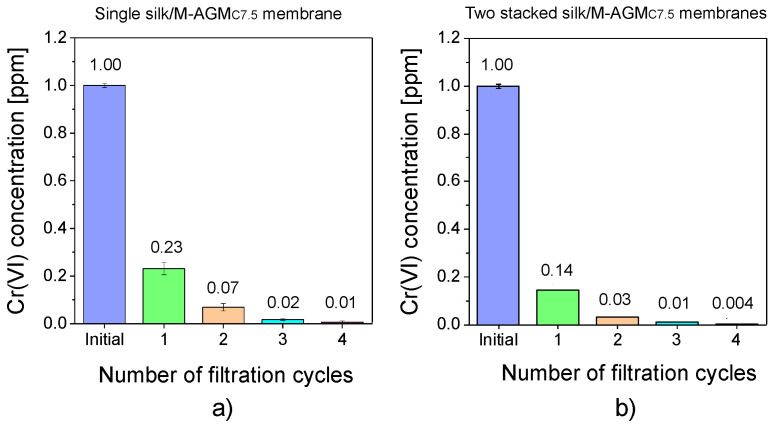
Results of the dynamic sorption tests carried out with 1 ppm Cr(VI) solutions: (**a**) single silk/M-AGMC7.5 membrane; (**b**) two stacked silk/M-AGMC7.5 membranes separated by a plastic net.

Dynamic sorption tests were performed on silk/M-AGM samples 2.5 and 7.5 cm in diameter by performing dead-end mode ultrafiltration experiments under a constant transmembrane pressure of 2.5 atm (Figure 9 and Figure 10). Depending on membrane size, ultrafiltration apparatuses of different capacities were used (50 mL for 2.5 cm diameter membranes and 600 mL for 7.5 cm diameter membranes). The ultrafiltration cells were filled with Cr(VI) aqueous solutions of known concentrations, that is, 30 mL of a 10 ppm Cr(VI) solution for 2.5 cm diameter membranes and 300 mL of a 1 ppm Cr(VI) solution for 7.5 cm diameter membranes. Silk/M-AGM membranes obtained using silk mats prepared by three different procedures (Table 1) and, therefore, characterized by different morphologies (Figure 6) were tested. Unsurprisingly, the three different membrane types exhibited different sorption capacities. In particular, the membranes prepared by method B were more compact and poorly permeable. As a result, they could not be adequately tested and were not studied further. The results obtained with the initial set of experiments carried out using 2.5 cm diameter membranes (silk/M-AGMA2.5 and silk/M-AGMC2.5) are shown in Figure 9. Two different setups were adopted, that is, the ultrafiltration apparatus was equipped with a single membrane (Figure 9c,d) or four stacked membranes separated from each other by a plastic mesh (Figure 9b,e). In addition, different filtration cycles were performed on the same aliquot of Cr(VI) solution. It may be noticed that in the case of silk/M-AGMA2.5 membranes, the residual Cr(VI) concentration in the filtrate only slightly reduced after each filtration cycle (Figure 9c), with an overall reduction after four passages of 26%. In the case of silk/M-AGMC2.5 membranes, the residual Cr(VI) concentration in the filtrate was reduced by 33% after a single cycle and steadily decreased after each filtration step down to 1.3 ppm Cr(VI) (a total reduction of 87%). It should be noticed that SEM analysis showed that the porosity of the silk/M-AGMA2.5 membranes was higher than that of the silk/M-AGMC2.5 membranes, while the porosity of the silk/M-AGMB2.5 membranes was very poor. Consequently, the flow rate measured in experiments carried out with silk/M-AGMC2.5 membranes was significantly lower than that in the presence of silk/M-AGMA2.5 membranes (0.3 vs. 1.0 cm^3^ s^−1^, respectively), while silk/M-AGMB2.5 membranes were not permeable. Consequently, in the case of silk/M-AGMC2.5 membranes, due to the contact time between the Cr(VI) ions and the active sites of the sorbent, Cr(VI) absorption efficiency was higher.

The results obtained with a second set of experiments carried out using 7.5 cm diameter membranes (silk/M-AGMC7.5) are shown in Figure 10. In this case, two different setups were also adopted: one with a single membrane (Figure 10a) and one with two stacked membranes separated by a plastic mesh (Figure 10b). It may be observed that a single membrane reduced Cr(VI) concentration down to 10 ppb, whereas two membranes reduced it down to 4 ppb, which is well below the threshold for drinkable water [7].

## 4. Conclusions

From the above results, the following conclusions can be drawn. First of all, the ability of silk mats obtained from short degummed raw silk fibers to reinforce PAA hydrogels has been widely confirmed. In particular, tough PAA-based membranes were obtained by first impregnating silk mats with an aqueous solution of an α,ω-bisacrylamide-terminated amphoteric PAA oligomer called M-AGM (prepared by the polyaddition of 4-aminobutylguanidine with excess *N,N’*-methylenebisacrylamide) and then triggering the radical polymerization of the M-AGM acrylamide terminals through UV irradiation.

It is reasonable to hypothesize that, during this process, covalent bonds are formed by the aza-Michael reaction of the amino groups of lysine present in silk with a part of the acrylamide end groups of M-AGM oligomers. This would explain the high dimensional stability of the composite membrane, which is characterized by high integration of the fibrous hydrogel matrix, even after prolonged incubation in water. The silk/M-AGM membranes were in fact reversibly swellable in water, and their bulk remained unaffected after several water uptake/drying cycles, showing no evidence of separation of its components.

SEM analyses confirmed an intimate interconnection between fibers and polymer matrix in the composite membranes, whose porosity levels depended on the specific preparation procedure adopted and, if opportune, could be purposely created.

The presence of guanidine pendants in the M-AGM repeat units imparted the ability to form strong but reversible interactions with chromate ions. This property was confirmed by performing sorption experiments with Cr(VI) aqueous solutions with concentrations from 20 to 1 ppm in both static and dynamic conditions, that is, either by incubating the silk/M-AGM membranes in 20–2.5 ppm Cr(VI) solutions or by ultrafiltering 10 and 1 ppm Cr(VI) solutions under a pressure of 2.5 atm.

After performing static sorption experiments in 10 ppm Cr(VI) solutions, a rather high concentration specifically chosen to demonstrate the sorption and regeneration capabilities of the silk/M-AGM membranes, they could be simply regenerated by treatment with a solution of 1 M sodium hydroxide.

In this work, the ultimate goal was to study the ability of silk/M-AGM membranes to reduce the amount of Cr(VI) in water to negligible amounts (<50 ppb) by determining their ability in relation to waters polluted by Cr(VI) and previously subjected to purification treatments suitable for industrial application. This goal was achieved by preparing silk/M-AGM membranes which, through ultrafiltration of aqueous solutions of 1 ppm Cr(VI), reduced chromium content to a final concentration of 4 ppb.

In conclusion, the synthetic method established in this paper provides a broadly applicable and easily scalable procedure for reinforcing PAA hydrogels endowed with useful properties using silk waste otherwise destined for disposal. The exceptional versatility of PAA combined with a reinforcing agent from renewable sources has proven to be suitable for the design of new materials with both suitable mechanical resistance and sufficient absorption capacity toward highly toxic inorganic water pollutants. More specifically, since the mechanism of action of the silk/M-AGM membranes designed for the removal of Cr(VI) from polluted water is based on the presence of guanidinium pendants in their structure and their ability to stably interact with oxyanions, it can be reasonably expected that such membranes could effectively remove different types of oxyanions, thus broadening their application range.

## Figures and Tables

**Table 1:** Characteristics of the dry silk/M-AGM membranes prepared by procedures A, B, and C.

Sample ^(a)^	Silk Mat Weight (mg)	Silk/M-AGM Membrane Weight (mg)	Weight Percentage of M-AGM in the Membrane (%)	Silk/M-AGM Membrane Thickness (μm)
silk/M-AGMA2.5 ^(b)^	45.8 ± 3.6	86.9 ± 6.3	47 ± 4	127 ± 12
silk/M-AGMB2.5 ^(c)^	33.1 ± 6.9	86.7 ± 12.3	61 ± 5	125 ± 9
silk/M-AGMB7.5 ^(c)^	367.2 ± 32.3	1086.5 ± 83.8	66 ± 4	143 ± 24
silk/M-AGMC2.5 ^(c)^	33.1 ± 6.9	56.4 ± 6.7	48 ± 6	125 ± 9
silk/M-AGMC7.5 ^(c)^	367.2 ± 32.3	737.3 ± 58.8	46 ± 5	143 ± 24

^(a)^ Subscripts indicate both the adopted preparation procedure (A, B, or C, see Section 2.5) and the membrane diameter (2.5 or 7.5 cm). ^(b)^ Membranes prepared from compressed silk mats. ^(c)^ Membranes prepared from uncompressed silk mats.

**Table 2:** Results of sorption tests carried out in static conditions on silk/M-AGMA2.5 membranes ^(a)^.

Cr(VI) Concentration before Sorption Test (ppm)	Cr(VI) Weight in the Starting Solution (mg)	Cr(VI) Concentration after Sorption Test (ppm)	Cr(VI) Weight in the Final Solution (mg)	Cr(VI) Removal (%)	Mass of Cr(VI) Sorbed for Mass Unit of Dry Weight of the M-AGM Matrix (mgmg^−1^)
20	100	7.7 ± 0.2	38.5 ± 1.2	62	1.4 ± 0.10
10	50	3.1 ± 0.2	15.3 ± 0.8	69	0.8 ± 0.09
5	25	2.5 ± 0.6	12.7 ± 3.0	50	0.4 ± 0.07
2.5	12.5	1.3 ± 0.2	6.7 ± 1.1	48	0.2 ± 0.04

^(a)^ All tests were performed on 5 mL Cr(VI) solutions.

## Data Availability

Not applicable.
